# Behavioral Disorganization or Frontal Dysfunction? A Case Review

**DOI:** 10.1192/j.eurpsy.2026.12135

**Published:** 2026-07-31

**Authors:** L. S. Rodriguez, M. M. Castello, A. A. Gutierrez, B. C. Yagüe, M. V. Barea, M. V. Barea

**Affiliations:** Psychiatry, SAS, Jaen, Spain

## Abstract

**Introduction:**

Schizophrenia is a chronic psychiatric disorder characterized by disturbances in perception, thought, and behavior. The prefrontal cortex is central in this pathophysiology, and its alterations correlate with executive dysfunction, negative symptoms, and behavioral disorganization, including socially inappropriate or sexually disinhibited actions.

Frontotemporal dementia (FTD) is characterized mainly by alterations in behavior, executive function, and language. It is a leading cause of dementia before 65 years of age and is often underdiagnosed due to overlap with psychiatric disorders. Clinically, is subdivided into behavioral variant (bvFTD) and primary progressive aphasia (PPA). Its onset is insidious, with progressive decline in cognition and behavior.

**Objectives:**

A 40-year-old institutionalized male with schizophrenia and type 2 diabetes presented with increasing problematic behaviors over one year, including self-injury, conflicts with caregivers, and recent sexual disinhibition. He was admitted for evaluation and stabilization.

**Methods:**

On examination, he was conscious, cooperative, and approachable, with sparse speech, cognitive impairment, and auditory hallucinations, but without affective blunting or suicidal ideation. Laboratory tests were unremarkable; clozapine levels were 244 ng/mL. Neuroimaging revealed mild bilateral frontoparietal cortical atrophy on CT and frontal hypometabolism with atrophy on FDG-PET/CT. Pharmacological adjustments included clozapine titration and valproic acid introduction, leading to gradual remission. Neurology evaluated possible bvFTD.

**Results:**

Differential diagnosis between chronic schizophrenia and FTD requires integration of clinical, neuropsychological, and neuroimaging data. Both involve frontal lobe dysfunction, explaining overlaps such as executive deficits and disinhibition. In schizophrenia, negative symptoms correlate with dorsolateral and medial prefrontal dysfunction and chronic hypofrontality, while FTD presents a neurodegenerative course with progressive decline in executive, language, and social domains. In this patient, absence of progressive cognitive deterioration, persistence of disorganized symptoms, and improvement with clozapine support attribution to schizophrenia rather than bvFTD.

**Conclusions:**

Although both conditions involve frontal lobe alterations, the neuropsychological and neurofunctional findings, together with the clinical trajectory, support the interpretation that the behavioral manifestations in this case correspond to the disorganization intrinsic to chronic schizophrenia. The clinical and neurobiological analysis reinforces that behavioral disorganization and sexual disinhibition in this patient do not correspond to an FTD process but rather form part of the spectrum of negative and disorganized symptoms of chronic schizophrenia, in the context of prefrontal dysfunction characterized by hypofrontality.

**Disclosure of Interest:**

None Declared

